# COVID-19 associated pulmonary aspergillosis in critically-ill patients – authors’ reply

**DOI:** 10.1186/s13613-024-01320-3

**Published:** 2024-07-15

**Authors:** Pierre Bay, Etienne Audureau, Slim Fourati, Nicolas de Prost

**Affiliations:** 1 Médecine Intensive RéanimationService de Médecine Intensive Réanimation, Assistance Publique-Hôpitaux de Paris), Hôpitaux Universitaires Henri Mondor, DMU Médecine, CHU Henri Mondor, CEDEX, 51, Av. de Lattre de Tassigny, Créteil, 94010 France; 2 Groupe de Recherche Clinique CARMAS, Université Paris-Est-Créteil (UPEC), Créteil, France; 3 Université Paris-Est-Créteil (UPEC), Créteil, France; 4 IMRB INSERM U955, Team « Viruses, Cancer », Hepatology, Créteil, France; 5 IMRB INSERM U955, Team CEpiA, Unité de Recherche Clinique, Créteil, France; 6 Department of Public Health, Hôpitaux Universitaires Henri Mondor, Assistance Publique – Hôpitaux de Paris (AP-HP), Créteil, France; 7 Department of Virology, Hôpitaux Universitaires Henri Mondor, Assistance Publique – Hôpitaux de Paris, Créteil, France

We thank Zeng and Zhou for their interest in our recently published article on COVID-19-associated pulmonary aspergillosis (CAPA) in the era of Delta and Omicron variants [1].

Firstly, we would like to apologize for the typographical errors and inaccuracies in the tables, which have now been rectified. The correct number of patients of female gender among CAPA patients was indeed 8, corresponding to a proportion of 28%.

Secondly, we fully agree with the remarks concerning the diagnostic strategy of CAPA. In our cohort, the CAPA diagnosis work-up was targeted rather than routinely performed (i.e., at the initiative of the attending clinician). As accurately pointed out by Zeng and Zhou, no data related to the number of patients who underwent CAPA testing - notably bronchoalveolar (BAL) lavage sampling - was provided in our study. Indeed, such variables were not routinely recorded for patients included in the SEVARVIR prospective cohort. To our knowledge, no systematic screening strategy for CAPA has demonstrated clear patient benefits as compared to a targeted approach. We acknowledge that reported CAPA prevalence heavily depends on the chosen strategy, as recently illustrated by Feys et al. [2]. Some authors advocate for an active CAPA diagnostic approach, including early BAL sampling [2]. However, we believe this approach may lead to overdiagnosis of CAPA, especially given the nonspecific diagnostic criteria that fail to distinguish colonization from invasive fungal infection. Interestingly, this same group reported a CAPA prevalence of 33% in a recent study involving 335 mechanically ventilated COVID-19 patients [3]. It is also crucial to consider the therapeutic implications of overdiagnosing CAPA. Indeed, CAPA diagnosis almost invariably leads to the prescription of antifungal therapy (as reported in our study), with associated costs and toxicity that cannot be overlooked. Lastly, a targeted (clinician-led) strategy appears particularly suited to the evolving clinical phenotype of COVID-19 patients requiring intensive care unit admission. In our cohort, over half of the patients did not require invasive mechanical ventilation, making systematic BAL sampling for non-intubated, respiratory-compromised patients challenging to implement. Interestingly, the prevalence reported in our study among intubated patients (*N* = 22/242, 9.1%) is in line with the prevalence reported by a French multicenter study conducted during the first epidemic wave [4], which employed systematic screening (*N* = 76/565, 15%). To sum up, the debate between systematic and targeted CAPA screening currently relies solely on expert opinion and requires prospective, randomized trials comparing these strategies and their net clinical benefits. The future likely entails risk stratification and personalized medicine, similarly to fungal risk management in hematology, with a better understanding of each patient’s unique risk profile [5].

Thirdly, we acknowledge significant heterogeneity in patient enrolment across centres and the comprehensiveness of patient inclusion, as is common in clinical studies. Nonetheless, the large number and diversity of centres strengthen the external validity of our study.

Fourthly, as noted, the lack of reported significant mortality difference according to CAPA status in our study could be explained by several factors, including insufficient statistical power, antifungals efficacy, or the true prognostic impact of CAPA (which is challenging to differentiate from inherent patient risk factors for CAPA development).

Lastly, while a prophylaxis strategy may seem appealing given the delayed onset of CAPA, no patient in the study received such prophylaxis to our knowledge, as no prospective study has yet demonstrated its benefit.

In conclusion, we fully align with Zeng and Zhou on the need for prospective studies comparing CAPA diagnostic and therapeutic strategies (both preventive and curative) in critically-ill COVID-19 patients. A risk-stratified approach based on patient outcome and clinical presentation appears most reasonable at present.

## Data Availability

Not applicable.

## Abbreviations

CAPA: COVID-19-associated pulmonary aspergillosis
